# Kneeling (Incomplete Knee) Presentation: A Single Case Report

**DOI:** 10.7759/cureus.85928

**Published:** 2025-06-13

**Authors:** Platon Machavariani, Nickolas Kintraia, Maia Rizvadze, Nato Metskhvarishvili, Ketevan Grigalashvili, Marina Merkviladze, Ketevan Chichua

**Affiliations:** 1 Department of Obstetrics and Gynecology, Department of Perinatology, Tbilisi State Medical University (TSMU) The First University Clinic, Tbilisi, GEO; 2 Department of Obstetrics and Gynecology, Tbilisi State Medical University (TSMU) The First University Clinic, Tbilisi, GEO

**Keywords:** breech presentation, kneeling presentation, knee presentation, labor management, vaginal breech delivery

## Abstract

The kneeling breech presentation is extremely rare, especially among the deliveries at term; the information about the management of this type of fetal lie is poor. Here we discuss a case of a 24-year-old primipara woman, at 37 weeks of gestation, who was admitted to the hospital with a fully dilated cervix and ruptured membranes. The pelvic examination revealed an incomplete kneeling breech presentation.

## Introduction

Breech presentations at term typically occur in 3% to 4% of all deliveries [1,2]. The three main types of breech presentation include 1) Frank breech: both hips are flexed, and both knees are extended so that the feet are adjacent to the head. 2) Complete breech: both hips and both knees are flexed. 3) Incomplete breech: one or both hips are not completely flexed. In non-frank breech presentations, one or both feet (or rarely one or both knees) may present before the buttocks in the birth canal-footling and kneeling presentations. In an incomplete breech position, one or both hips are not completely flexed. This is the double-footling breech presentation. The same could be said about the kneeling position of the breech-presenting babies. If one knee presents, this is an incomplete kneeling; if both knees present, it is a complete kneeling presentation. Frank breech presentation accounts for 50 to 70% of breech fetuses at term, complete breech presentation accounts for 5 to 10% of breech fetuses at term, and incomplete breech presentation accounts for 10 to 40% of breech fetuses at term.

Breech presentations increase the potential for significant morbidity and mortality to both the mother and fetus [3-5].

## Case presentation

**Figure 1:**
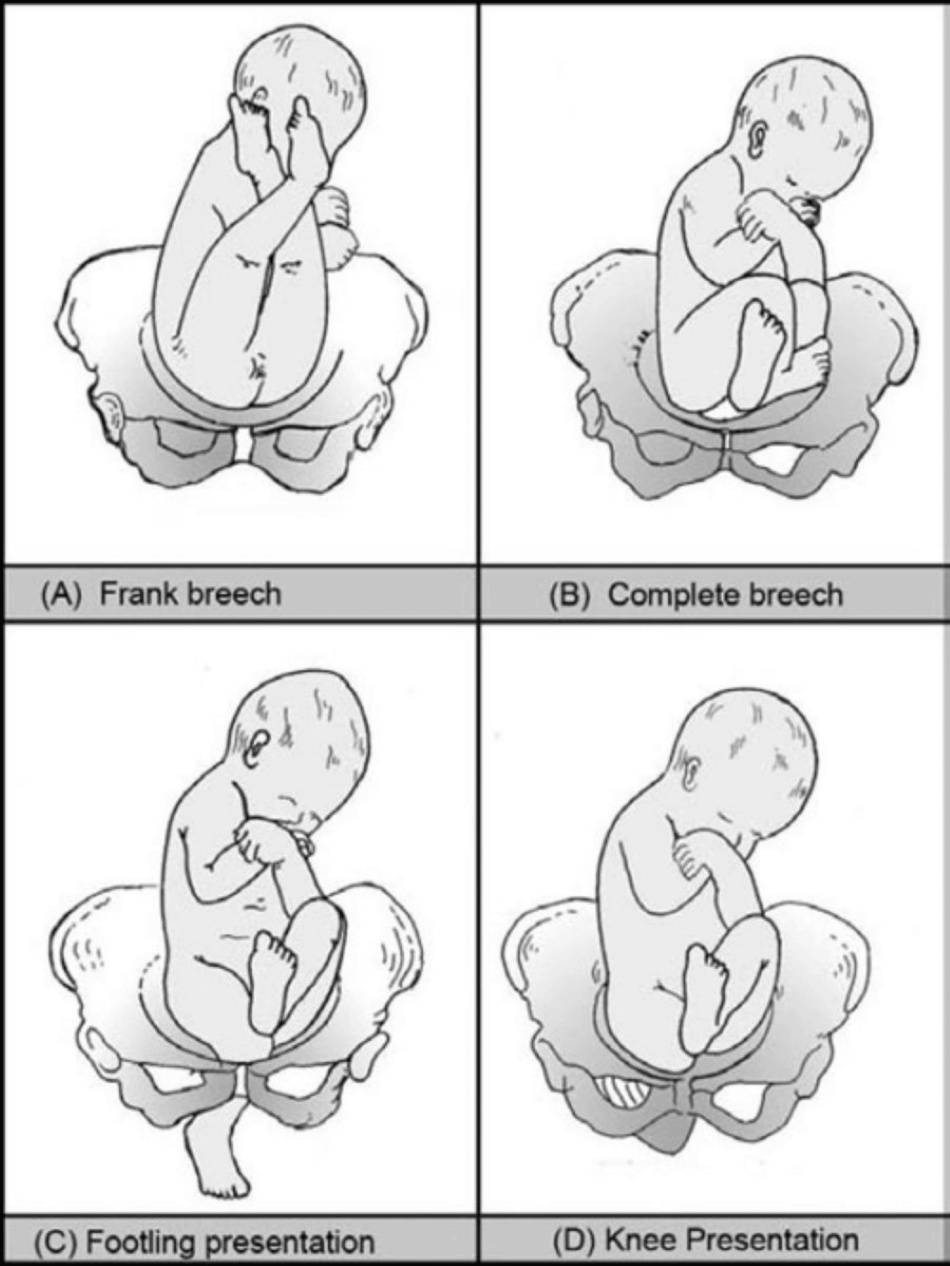
Footling and knee presentation This image is from Chapter 15 Breech Presentation of Manual of Midwifery by Jacob Annamma [6].

**Figure 2:**
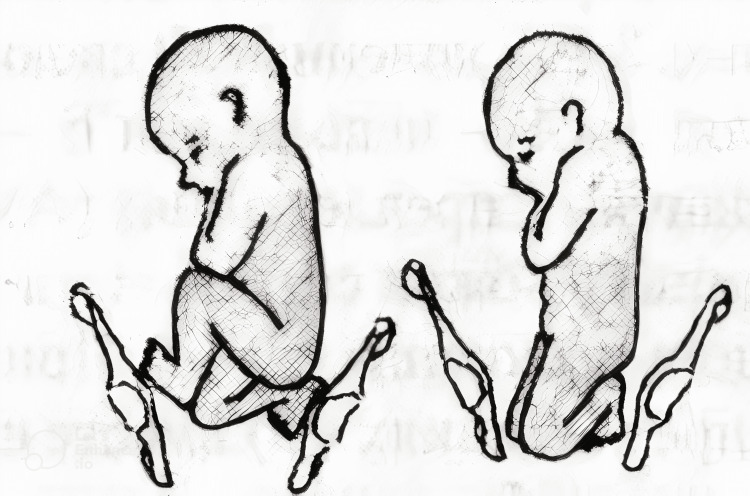
Complete and incomplete knee presentation. This image is a free image available at StudFiles' website [7].

**Figure 3:**
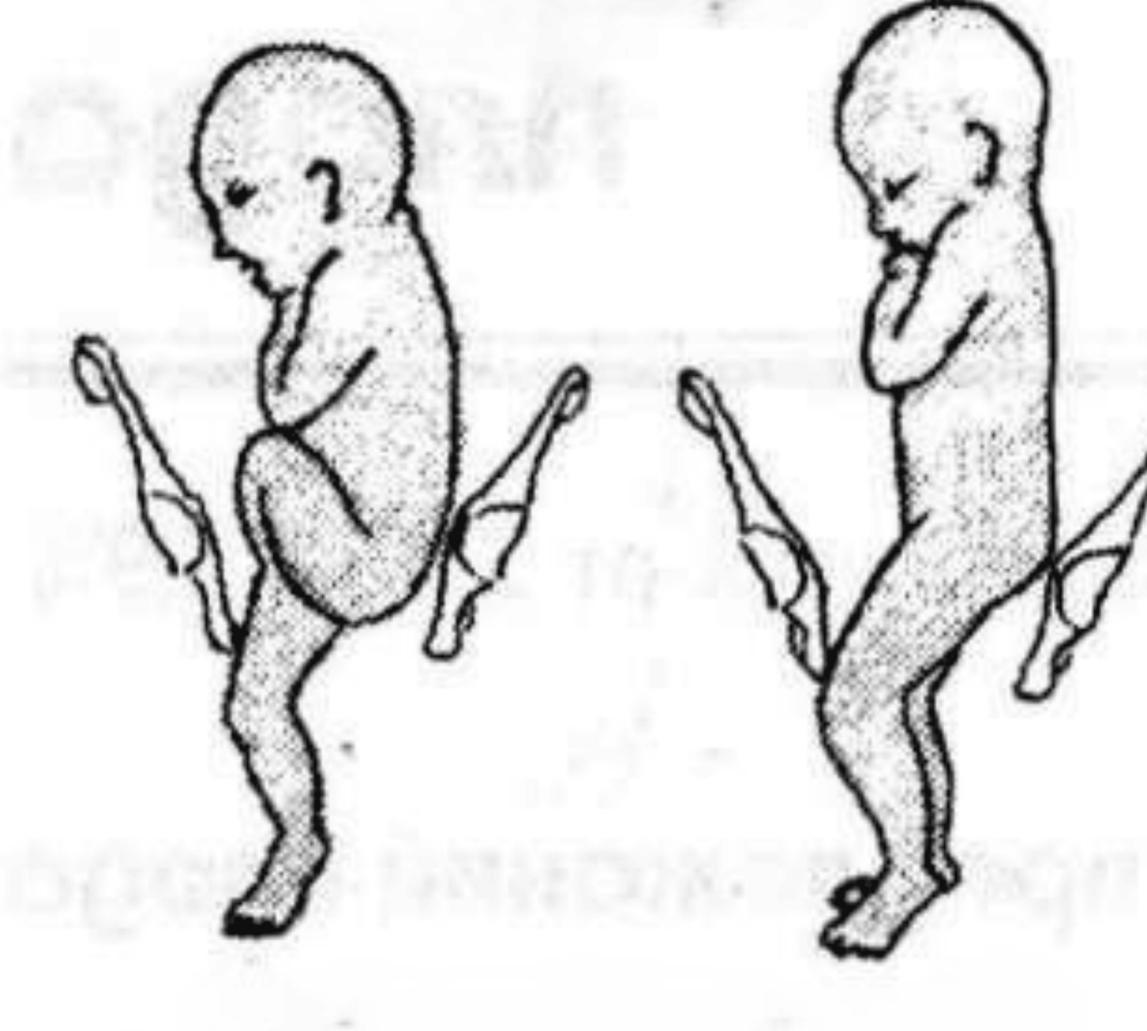
Complete and incomplete footling presentation This image is a free image available at StudFiles' website [7].

A 24-year-old primipara woman, at 37 weeks of gestation, was admitted to the hospital with a fully dilated cervix and ruptured membranes in the second period of labour, with bearing-down efforts every two minutes, with a duration of 50 seconds. The patient's BMI was 23.7 kg/m², height 168cm, and weight 67kg. The pelvic examination revealed an incomplete kneeling breech presentation. The knee was the lowest in the birth canal at the pelvic outlet. The fetal heart rate was 135-140 bpm with continuous CTG monitoring. The estimated weight of the fetus was 3300,0g ±200g. Although the kneeling presentation is an indication of cesarean delivery, at the admission parturient was in the second period of labour with regular, effective bearing-down efforts, and vaginal examination revealed a single knee at the outlet of the bony pelvis; the fetal heart rate was in a normal range. Under the supervision of a senior obstetrician, the decision of vaginal delivery was made. The patient was fully informed about the risks associated with vaginal breech delivery. The maternal bearing-down efforts resulted in the expulsion of the fetus at the umbilicus level. At this time, suprapubic pressure was applied to promote head flexion. With ongoing bearing down, the trunk was delivered at the scapula level, supported by the operator. The shoulders were delivered spontaneously along with the arms. The delivery of the head was assisted using the Mauriceau-Smellie-Veit manoeuvre. The female newborn was delivered with a mass of 3200.0g, a length of 50cm, and an Apgar score of 8/9 at 1/5 minutes, respectively. There were no neonatal complications, such as fractures, following delivery or the neonatal intensive care unit (NICU) admission. The third period of labour was managed actively. The placenta was delivered within 5 minutes; the uterus contracted well. There was a first-degree perineal tear, which was subsequently sutured typically (Figures 1-3).

## Discussion

Breech presentations at term typically occur in 3% to 4% of all deliveries of singleton term breech fetuses [1,2]. Kneeling presentation, also known as a rare variant of breech presentation, involves the fetus presenting with knees, resulting in the knees being the leading part of the birth canal. This presentation is uncommon and poses specific challenges for vaginal delivery, increasing the risk of complications such as cord prolapse and birth trauma [8]. This type of presentation increases the potential for significant morbidity and mortality to both the mother and fetus [3-5]. Although this specific presentation is infrequently addressed directly in clinical guidelines, both the American College of Obstetricians and Gynecologists (ACOG) and the Royal College of Obstetricians and Gynaecologists (RCOG) include it under broader recommendations for breech management [9,10]. Due to the atypical position, the presenting knees may not adequately dilate the cervix or engage the pelvis, leading to the risk of obstructed labour. As a result, cesarean section is often the recommended mode of delivery [8]. Management strategies during a kneeling presentation may be limited due to its rarity [9,10].

According to ACOG, breech presentations should generally be delivered via cesarean section due to increased neonatal morbidity and mortality associated with vaginal breech delivery, particularly when criteria for safe vaginal delivery are not met [9]. While the ACOG guideline does not explicitly mention kneeling presentation, the management falls under non-frank breech presentations, which are generally contraindications for vaginal delivery unless under specific controlled conditions [9]. Planned cesarean delivery is recommended for term singleton breech presentations, especially if the type is incomplete (footling or kneeling). Vaginal breech delivery may be considered only in selected candidates with frank or complete breech, a clinician experienced in breech delivery, an adequate pelvis, no fetal anomalies or growth restriction [9], an absence of coexistent pregnancy complications, patient preference, hospital capabilities, fetal size, anatomy, and gestational age [11]. With hyperextension, vaginal delivery can injure the fetal spinal cord. Thus, if identified at term, cesarean delivery is indicated [12]. However, cases of spinal cord injury have been reported following uneventful cesarean delivery of breech fetuses. Here, the flexion itself may be implicated [13,14]. RCOG also considers kneeling presentation a contraindication for vaginal breech birth, given its association with higher complication rates, and advises cesarean section as the safer mode of delivery in such cases (10). ACOG and RCOG both endorse the external cephalic version (ECV), which is between 36 and 37 weeks without contraindications, though ECV may be less effective in atypical presentations like kneeling [9,10]. Vaginal breech delivery may be considered in selected cases of frank or complete breech [10].

Mainly, a fetus presenting in the kneeling position is an indication of a C-section. But, sometimes, the parturient can present with full cervical dilation and the fetal extremities at the pelvic outlet. Occurs the question of what has the priority: c-section or vaginal delivery?

## Conclusions

The kneeling breech presentation is rare, especially among deliveries at term; the information about the management of this type of fetal lie is poor. Breech presentations, especially the footling and kneeling breech presentations, increase the potential for significant morbidity and mortality to both the mother and fetus. Since the parturient was admitted with a fully dilated cervix and ruptured membranes in the second period of labour, and the presenting part of the fetus was a single knee located at the pelvic outlet, with good intensity of the pushing effort, the decision was made to deliver the fetus vaginally under continuous Cardiotocography (CTG) monitoring. Although the case of kneeling presentation is an indication for cesarean delivery, adequate maternal pushing effort with normal fetal CTG results, normal fetal size, and location of knees at the pelvic outlet was the reason to terminate delivery vaginally, under the supervision of a senior obstetrician.
